# Concurrent use of alcohol interactive medications and alcohol in older adults: a systematic review of prevalence and associated adverse outcomes

**DOI:** 10.1186/s12877-017-0532-2

**Published:** 2017-07-17

**Authors:** Alice E. Holton, Paul Gallagher, Tom Fahey, Gráinne Cousins

**Affiliations:** 1 School of Pharmacy, Royal College of Surgeons in Ireland (RCSI), Dublin 2, Ireland; 2 HRB Centre for Primary Care Research, Department of General Practice, Royal College of Surgeons in Ireland (RCSI), Dublin 2, Ireland

**Keywords:** Alcohol, Drug interaction, Alcohol interactive, Older adult, Psychotropic medicines, Adverse outcomes

## Abstract

**Background:**

Older adults are susceptible to adverse effects from the concurrent use of medications and alcohol. The aim of this study was to systematically review the prevalence of concurrent use of alcohol and alcohol-interactive (AI) medicines in older adults and associated adverse outcomes.

**Methods:**

A systematic search was performed using MEDLINE (PubMed), Embase, Scopus and Web of Science (January 1990 to June 2016), and hand searching references of retrieved articles. Observational studies reporting on the concurrent use of alcohol and AI medicines in the same or overlapping recall periods in older adults were included. Two independent reviewers verified that studies met the inclusion criteria, critically appraised included studies and extracted relevant data. A narrative synthesis is provided.

**Results:**

Twenty studies, all cross-sectional, were included. Nine studies classified a wide range of medicines as AI using different medication compendia, thus resulting in heterogeneity across studies. Three studies investigated any medication use and eight focused on psychotropic medications. Based on the quality assessment of included studies, the most reliable estimate of concurrent use in older adults ranges between 21 and 35%. The most reliable estimate of concurrent use of psychotropic medications and alcohol ranges between 7.4 and 7.75%. No study examined longitudinal associations with adverse outcomes. Three cross-sectional studies reported on falls with mixed findings, while one study reported on the association between moderate alcohol consumption and adverse drug reactions at hospital admission.

**Conclusions:**

While there appears to be a high propensity for alcohol-medication interactions in older adults, there is a lack of consensus regarding what constitutes an AI medication. An explicit list of AI medications needs to be derived and validated prospectively to quantify the magnitude of risk posed by the concurrent use of alcohol for adverse outcomes in older adults. This will allow for risk stratification of older adults at the point of prescribing, and prioritise alcohol screening and brief alcohol interventions in high-risk groups.

**Electronic supplementary material:**

The online version of this article (doi:10.1186/s12877-017-0532-2) contains supplementary material, which is available to authorized users.

## Background

By 2050, older adults aged ≥60 years are expected to account for 34% of the population in Europe [1]. While alcohol consumption changes over the life-course, with a decline in consumption in older age, recent evidence from nine UK based prospective cohort studies, have shown that drinking occasions tend to become more frequent among older adults [2]. There is also evidence of a cohort effect, with successive birth cohorts reporting an increase in alcohol consumption across all age-groups, including among older adults [3].

Even at relatively low levels of alcohol consumption, older adults can be vulnerable to harm, with physiological changes exacerbating these harms [4, 5]. Furthermore, older adults experience a disproportionate burden of alcohol related-harm; in England between 2009 and 2010, adults aged ≥65 years accounted for approximately 44% (461,400) of alcohol-related hospital admissions yet comprised of only 17% of the population [6, 7]. Alcohol-related deaths were also highest among those aged 55 to 74 years [4].

Furthermore, the use of multiple medicines is increasing in older adults [8–11]. A recent Irish study reported an increase in polypharmacy from 17.8 to 60.4% between the years 1997–2012 in older adults aged ≥65 years [11]. Certain medications have the potential to interact with alcohol; these medications are referred to as alcohol interactive (AI) medications [12]. They may interact with alcohol by altering the metabolism (pharmacokinetic) or effects (pharmacodynamic) of alcohol and/or the medication [12]. Certain interactions may occur with any alcohol consumption, whereas other interactions may follow a dose response, with the risk or severity of an interaction increasing with increasing levels of alcohol [13]. AI medications, when combined with alcohol, increase the risk of medical complications such as hypoglycaemia, hypotension, sedation, gastrointestinal bleeds and liver damage, in older adults [5, 12]. For example, older adults are vulnerable to the sedating effects of alcohol and when combined with central nervous system (CNS) agents, such as psychotropic medications, older adults have an increased risk of sedation and drowsiness [5]. Psychotropic medicines include antidepressants, sedatives/hypnotics, stimulants and neuroleptics, all of which act on the CNS and are commonly prescribed to older adults [14]. Similarly, concurrent use of alcohol with cardiovascular agents, such as vasodilatory agents, increases the risk of hypotension in older adults [5], with concurrent use with non-steroidal anti-inflammatory drugs (NSAIDs) increasing the risk of gastrointestinal bleeds [12].

While a recent systematic review has assessed the prevalence of concurrent alcohol use and prescription sedative-hypnotic medicines in middle-aged and older adults, [15] there have been no systematic reviews on the prevalence of concurrent use of alcohol and alcohol interactive medications beyond psychotropic medications and associated adverse outcomes in older adults. An older review did investigate the potential risk of combining alcohol with medications in older adults however the focus of the review was on the pharmacology and mechanism of action involved in alcohol-medication interactions and potential clinical implications of these interactions [5]. Therefore this study aims to systematically review the prevalence of concurrent use of alcohol and alcohol interactive medications in older adults and associated adverse outcomes.

## Methods

This systematic review was performed according to Preferred Reporting Items for Systematic reviews and Meta-Analyses (PRISMA) guidelines [16].

### Search strategy

A comprehensive systematic search was performed using MEDLINE (PubMed), Embase, Scopus and Web of Science. A combination of the following keywords and MeSH terms were used: “ethanol”, “alcohol”, “drug interactions”, “drug alcohol interaction” and “aged”. This search was supplemented by a search in Google Scholar and by hand searching references of retrieved articles. The search was restricted to English language articles and articles published since January 1990 to June 2016.

### Study selection and data extraction

Studies were included if they met the following eligibility criteria: Observational studies reporting on the concurrent use of alcohol and alcohol interactive (AI) medicines in the same or overlapping recall periods in older adults. Studies also had to report on the quantity or frequency of alcohol consumption. We excluded studies which exclusively sampled patients with specific illnesses, or those seeking treatment for alcohol use disorders (AUD) or illicit drug use.

Title and abstracts of identified studies were reviewed by one reviewer (AH) to determine potential eligibility. Full text articles were then reviewed by two reviewers (AH/GC) for those studies considered eligible from title/abstract, or when it was unclear whether a study met the inclusion criteria. The following data were extracted by two reviewers (AH/GC): year of publication, country, study sample, study design, measurement and definition of alcohol interactive (AI) medications, measurement of alcohol consumption, prevalence of alcohol use and AI medication use and prevalence of concurrent alcohol and AI medication use. Adverse outcomes associated with concurrent use of alcohol and AI medications were also extracted if reported. Any uncertainty in relation to study eligibility and data extraction was resolved through discussion between two reviewers (GC/AH).

### Critical appraisal

The risk of bias was evaluated, by two reviewers (AH/GC), using an adapted form of the Newcastle Ottawa cohort scale (NOS) [17]. This amended NOS scale allowed for the evaluation of cross-sectional studies, focussing on the risk of selection bias and information bias, specifically misclassification bias of both exposures (alcohol and AI medications) and the outcome (concurrent use of alcohol and AI medications) across included studies.

## Results

**Fig. 1:**
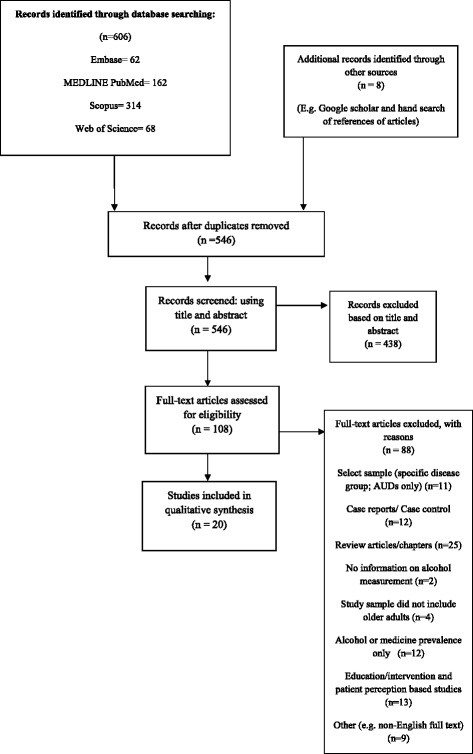
Flow diagram of studies included in this review

Of the 546 citations identified from this search strategy, 108 full text articles were assessed for eligibility, with 20 studies meeting the inclusion criteria [14, 18–36] (Fig. 1).

### Study characteristics

Ten of the included studies were conducted in Europe [14, 19, 21–23, 25, 27–29, 36], eight in North America [18, 20, 24, 30–32, 34, 35] and two in Australia [26, 33]. (Table 1) All studies were cross-sectional [14, 18–36]. Study settings varied across studies with; community-dwelling [14, 19, 21–27, 31, 34–36], both community dwelling or living independently in care facilities [32], general populations [20, 28, 33], hospital setting [29], retirement communities [18] and participants signed up to a pharmaceutical assistance contract for the elderly [30] reported.

**Table 1:** Characteristics of included studies

Reference	Setting	Participants: N, sex, mean age (±sd), range	Study design (survey mode)	Measurement &Definition:	
				AI Medication(s)	Alcohol Consumption Quantity/Frequency; thresholds applied
Adams 1995 [18]	United States (US), retirement community residents	*N* = 311 23% men 83 (± 6 years) NR	Cross sectional (Mailed self-reported survey)	Regular or occasional use of “high risk” meds in last 6 months: NSAIDS, aspirin, sedatives, narcotics, antidepressants, anti-hypertensives, antacids, H_2_ blockers, warfarin & meds for congestive heart failure, gout or diabetes. Reference source not reported	Khavari questionnaire: quantity – frequency, last 6 months, None, 1–6 drinks/week or ≥7 drinks/week 1 drink = 12 oz. of beer, 5 oz. of wine, 3 oz. of fortified wine or 1.5 oz. of hard liquor.
Aira 2005 [19]	Finland, community dwelling older adults	*N* = 521 27% men 81 (± 4.4 years) Range: 75–95.7 years	Cross sectional (Nurse interview; prescriptions & containers)	Current use of medications having potential interactions with alcohol: Acetaminophen (Paracetamol), anticonvulsants, antidepressants, TCAs, antihistamines, benzodiazepines, H_2_ receptor antagonists, neuroleptics, nitrates, NSAIDs, opiates & warfarin. Reference source not reported	Beverage specific quantity-frequency, last 12 months: None, 1–7 units/week, >7 units /week 1unit = 11-12 g of alcohol
Breslow 2015 [20]	US, general population ≥ 20 years	≥65 years *N* = 7183 ≥20 years *N* = 26,657 51% men NR	Cross sectional (Interviews in-home, medication containers)	Use of AI prescription medication, past month; identified using (i) Drugs.com, (ii) Caremark.com, (iii) Healthline.com, (iv) DailyMed databases & (v) references from 3 publications [12, 43, 44] including: cardiovascular agents, CNS agents, coagulation modifiers, GI, metabolic, psychotherapeutic & respiratory agents	Quantity – frequency in last 12 months: None, for women of all ages & men >65 years: moderate consumption: >0–7 drinks/week & heavier drinkers (> 7 drinks/week). For men 20–64 years moderate: >0–14 drinks/week & heavier >14drinks/week
Cousins 2013 [21]	Ireland, community dwelling older adults ≥60 years	*N* = 3815 46.6% males 69.7 (±7.3 years) Range: 60–99 years	Cross sectional (Nurse led interviews in-home; medications reviewed & self-completed questionnaire)	Current or regular use of medications with potential to interact with alcohol identified using Stockley’s Drug Interactions, British National Formulary and Irish Medicines Formulary: cardiovascular agents, CNS agents, antihistamines, blood agents, antidiabetic agents, anti-infectives, GI drugs, immunomodulators & muscle relaxants	Quantity-frequency, past 6 months: None, Light/moderate (≤ 4 drinks/day or 10 drinks/ week) and heavy drinkers (>4 drinks/day or 10 drinks/ week) 1 drink = 10 g of alcohol
Del Rio 1996 [23]	Spain, community dwelling adults ≥ 16 years	≥66 years *N* = 3003 ≥ 16 years *N* = 21,084 48% men NR	Cross sectional (survey with interview)	Medication use, past 2 weeks. No reference source reported	Beverage specific quantity-frequency, past 2 weeks
Del Rio 2002 [22]	Spain, community dwelling adults ≥ 16 years	≥66 years = 1025 ≥16 years *N* = 6396 48% men NR	Cross sectional (survey with interview)	Benzodiazepine use, past 2 weeks	Beverage specific quantity-frequency past 2 weeks: Low (men: ≤ 21 units/week, women: ≤ 14 units/week), moderate (men: 22–50 units/week, women: 15–35 units/week) & high consumption (men: >50 units/week & women: >35 units/week) 1 unit = 10 g of alcohol
Du 2008 [14]	Germany, community dwelling older adults	*N* = 1605 45.2% men NR Range: 60–79 years	Cross sectional (survey & interview; prescriptions or original containers)	Any psychotropic medication use in past 7 days: ATC nervous system drugs coded N00. (Excluded drugs coded N02B aspirin & paracetamol, except for N02BA71) Opiate codeines used as antitussives were merged with N02A & opiates for GI conditions (A07D) were not recorded	Beverage specific quantity-frequency, past 12 months: Problem use (risky drinking): daily consumption ≥10 g for women & ≥ 20 g for men
Forster 1993 [24]	US, community dwelling older adults	*N*=667 39.9% men 74.1 (±6.6 years) NR	Cross sectional (survey & interview; medication containers)	Prescription or OTC medications currently or past month: AI drugs defined by study clinical team: OTC painkillers, anti-hypertensives, diuretics, OTC cold preparations, arthritis medications, heart medications, antibiotics, mind altering medications, chest pain medications, pain medications, diabetes medications, ulcer medications, sleeping pills, OTC nasal sprays, steroids, blood agents, insulin, seizure medications & OTC asthma medication	Frequency: never, rarely, sometimes & regularly
Ilomaki 2008 [25]	Finland, community dwelling older adults	*N*=1,774 48.1% men 63 years (NR) Range: 53-73 years	Cross sectional (mailed survey & interview; prescriptions brought if any problems)	Regular use of psychotropic drugs at time of interview: antipsychotics, anxiolytics, hypnotics, sedatives, antidepressants & combinations	Beverage specific quantity-frequency past 12 months: frequent ≥2 times/week, binge (men ≥5 units/occasion: women ≥4 units/occasion) & heavy drinking (men: >14 units/week: women >7 units/week 1 unit=12 g of alcohol
Ilomaki 2013 [26]	Australia, community dwelling older men	≥70 years N=1,705 100% men NR NR	Cross sectional (Interview: medications brought)	Antidepressants (including SSRIS, TCAs, MAOIs and others) & SADs (including benzodiazepines and benzodiazepine-like hypnotics)	Beverage specific quantity-frequency past 12 months: daily (7days/week), binge (≥5 drinks at least once/month), heavy (>2 drinks/day) & problem drinkers (CAGE score ≥2). Nondrinkers: former or never drinkers 1 drink=10 g of alcohol
Immonen 2012 [27]	Finland, community dwelling older adults	≥ 65 years N=1,395 35.5% men 78.7 years (NR) NR	Cross sectional (mailed survey)	Current prescribed medications: Swedish, Finnish Interaction X-referencing (SFINX) interaction database identified significant drug-alcohol interactions: metronidazole, tinidazole, disulfiram, griseofulvin, prazosin, metformin & tacrolimus. CNS agents, hypoglycaemics and warfarin	Beverage specific quantity-frequency: At risk (>7 drinks/ week, or ≥5 drinks on a typical drinking day or ≥3 drinks several times/week), moderate drinkers (at least 1 drink/month but < 7 drinks/week) & minimal/non-users (<1 drink/month) 1 drink = 12 g of alcohol
John 2007 [28]	Germany, general population ≥ 20 years	NR ≥20 years *N*= 4290 49% men NR Range: 20-79 years	Cross sectional; (Interview; container or participant provided information on current medications)	Use of sedative, hypnotics or anxiolytics (SHA) medicines including: barbiturates (& derivatives), benzodiazepines (& derivatives), carbamates, piperidinedione derivatives, hypnotics or anxiolytics & combinations of sedatives and hypnotics. SO group: included SHA and opioid, past 7 days	Quantity of beverage specific alcohol consumed on last working day and last weekend: Risk drinking (men: >30 g/day & women >20g/day)
Lagnaoui 2001 [36]	France, community dwelling older adults	≥65 years *N*=3767 41.7% men NR NR	Cross sectional; (Interview at home; adhoc questionnaire; visual inspection)	Benzodiazepine use	Quantity: Daily wine consumption divided into: I) none ii) moderate up to 0.25 l day ^-1^ & iii) heavy > 0.25 l day ^-1^ ( l day^−1^= litres consumed daily)
Onder 2002 [29]	Italy, hospital admissions among older adults	*N* = 22,778 45% men 70.3 (± 16 years) NR	Cross sectional (questionnaire at admissions)	Medications taken prior to admission, during hospital stay & prescribed at discharge: drugs classified according to risk of causing adverse drug reactions using Naranjo algorithm	Quantity of daily consumption prior to hospital admission: (daily wine units). Non-drinkers & moderate drinkers only, heavy alcohol users were excluded
Pringle 2005 [30]	US, Pennsylvania Pharmaceutical Assistance Contract for the Elderly prescription drug users	*N* = 83,321 19% men 78.8 (± 6.9 years) Range: 65-106 years	Cross sectional (survey & prescription claims data)	Filled prescriptions, past 45 days: drugs with potential for alcohol interactions (using First Databank Inc. Drugs) with one of the following warnings considered AI: 1. May cause drowsiness; alcohol may intensify this effect 2. Do not drink alcohol when taking this medication 3. May cause drowsiness and dizziness; alcohol may intensify this effect 4. Limit alcohol while taking this medication; daily use of alcohol may increase risk of stomach bleeds	Current quantity-frequency: Light: 1-7 drinks/month Moderate: 8-30 drinks/month Heavy: >30 drinks/month
Qato 2015 [31]	US, community dwelling older adults	*N*= 2975 48.6% men NR Range: 57-85 years	Cross sectional (In house interviews & observation medication bottles)	Current or regular use (daily or weekly) with potential to interact with alcohol (Thomson Micromedex database). Drugs categorised according to severity of interaction: 1. Contraindicated: Drugs contraindicated for use with alcohol 2. Major: may be life-threatening or require medical intervention to prevent serious adverse events 3. Moderate: may result in exacerbation of individuals condition or require alternative therapy 4. Minor: limited clinical effects	Quantity-frequency last 3 months: Non-regular drinkers: (none or <1 drink/week) Light –regular: (≥ 1 drink/week & 1 drink/day) Heavy-regular: (≥ 1 drink/week & 2-3 drinks/day) Binge-regular: (≥ 1 drink/week & ≥4 drinks/day)
Sheahan 1995 [32]	US, community dwelling & independent living congregate care facility older residents	≥ 55 years *N*=1028 26.9% men 73.5 (± 9.47 years) NR	Cross sectional (In house interviews & medication container labels)	Psychotropic medications in past year: sedatives/hypnotics, anxiolytics/tranquilizers, antidepressants & prescription pain medications	Frequency, past year. Average number of times/week; month or year consumed alcohol. Number of drinking days in past year calculated
Veldhuizen 2009 [34]	Canada, community dwelling ≥ 15 years	NR ≥15 years: *N*=36,984 49% men N	Cross sectional study (Survey with interview, medication containers)	Use of benzodiazepines (N03AE, N05BA and N05CD), non-benzodiazepine hypnotics (zopiclone, eszopiclone, zolpidem and zaleplon) during past 2 days among those who reported use in past 12 months	Quantity-frequency on each of the previous 7 days among those drinking in past year: No drinking, moderate & heavy drinking (>14 drinks/week for men/ >9 drinks/week for women) or binge drinking (>4 drinks/day for women or 5 drinks/day for men) 1 drink= 1 bottle or can of beer/ glass of draft, 1 glass of wine/cooler or 1 ½ oz of liquor
Wong 2016 [35]	America, community dwelling older adults	*N*=2444 33% men 76.84 (±8.13 years) Range: 60-103 years	Cross sectional (Face to face interviews)	Prescriptions and OTC medications taken daily. Number of medications: low ≤ 1, moderate 2-4 & high ≥5	Quantity-frequency Abstainers: no to alcohol & 0 drinkers/month, Light: yes to alcohol & <29 drinks/month, Moderate: yes to alcohol & 30 drinks/month, Heavy: yes to alcohol & >31 drinks/month 1 drink= 14g of alcohol

Sample sizes varied from 311 to 83,321 participants [18, 30] . Men and women were included in all studies, with the exception of one Australian study which only included men [26]. Nine studies reported on a wide range of prescription and/or over the counter (OTC) medicines with potential to interact with alcohol [18–21, 24, 27, 29–31] . Three studies investigated any medication use during the recall period [23, 33, 35], and a further eight focused on psychotropic medications [14, 22, 25, 26, 28, 32, 34, 36]. Of the eight studies focusing on psychotropic medications, five investigated psychotropic medications alone [22, 25, 26, 34, 36] and three studies also included analgesics [14, 28, 32].

Of the nine studies focusing on a wide range of AI medications, all studies classified central nervous system (CNS) agents as AI medications (Table 1). Consistent with those studies investigating psychotropic medications [14, 22, 25, 26, 28, 32, 34, 36], the following drug classes were classified as AI medications, sedatives/hypnotics [18–21, 24, 27, 30, 31], antidepressants [18–21, 27, 30, 31], opioids/narcotics [18–21, 27, 30, 31], anticonvulsants [19–21, 24, 27, 30, 31] and anti-psychotics [19–21, 27, 29, 30]. After CNS agents, cardiovascular medicines (CVS) were the most common AI medicines [18–21, 24, 27, 29–31], followed by antidiabetic drugs [18, 20, 21, 24, 27, 29–31], warfarin [18–21, 24, 27, 30, 31], gastrointestinal agents [18–21, 24, 30, 31], non-steroidal anti-inflammatory drugs (NSAIDs) [18–21, 29–31], antibiotics/anti-infectives [21, 24, 27, 29–31] and anti-histamines [19–21, 30].

### Quality assessment

The methodological quality of the included studies is detailed in Table 2. The external validity was high in 4 studies, as they reported on random population samples of community dwelling older adults aged ≥60 or ≥65 years [21, 24, 27] or in the case of Breslow et al., a random population sample with oversampling of older adults aged ≥60 years [20]. Ten studies were considered to have moderate external validity; five studies sampled random community dwelling older adults but with age restrictions which may have introduced selection bias [14, 19, 25, 26, 31] such as including adults aged between 53 and 75 years [25] or only including adults ≥75 years [19]. A further five studies reported on random population samples of community dwelling adults with subgroup analysis of older adults, however they did not report oversampling of older adults [22, 23, 28, 33, 34]. The six remaining studies were considered to have poor external validity, as the risk of selection bias is considered to be high [18, 29, 30, 32, 35, 36]. For example, Pringle et al. recruited enrollees in the Pennsylvania pharmaceutical assistance contract for the elderly, which may not be representative of all older adults as the members are older (mean age 78.8 years) and more likely to be female and white with multiple chronic conditions [30].

**Table 2:** Results of the critical appraisal of included studies

Study	Representativeness of sample	Ascertainment of alcohol consumption	Ascertainment of AI medications (classified)	Ascertainment of AI medications (measured)	Assessment of the outcome (concurrent use)	Study total:
a) Cross Sectional Studies Outcome of Interest Concurrent use of alcohol and medications:						
Adams et al. 1995 [18]		++	+			+++
Aira et al. 2005 [19]	+	++		++		+++++
Breslow et al. 2015 [20]	++	++	++	++	++	++++++++++
Cousins et al. 2014 [21]	++	++	++	++	++	++++++++++
Del Rio et al. 1996 [23]	+	++				+++
Del Rio et al. 2002 [22]	+	++	++			+++++
Du et al. 2008 [14]	+	++	++	++	++	+++++++++
Forster et al. 1995 [24]	++	+		++		+++++
Ilomaki et al. 2008 [25]	+	++	++	++	++	+++++++++
Ilomaki et al. 2013 [26]	+	++	++	++	++	+++++++++
Immonen et al. 2012 [27]	++	+	++			+++++
John et al. 2007 [28]	+	+	++	++		++++++
Lagnaoui et al. 2001 [36]			++			++
Onder et al. 2002 [29]		+				+
Pringle et al. 2005 [30]		+	++	++		+++++
Sheahan et al. 1995 [32]		+	++	++		+++++
Qato et al. 2015 [31]	+	++	++	++	++	+++++++++
Swift et al. 2007 [33]	+					+
Veldhuizen et al. 2009 [34]	+	++	++	++	++	+++++++++
Wong et al. 2016 [35]						

Internal validity was assessed by evaluating the potential risk of misclassification bias for both exposure and outcome across studies. Ten studies were considered to have a low risk of misclassifying exposure to medications which have the potential to interact with alcohol, as they used prescription claims data or in house inventories where they recorded details from the labels of medication containers or prescriptions and provided references supporting the inclusion of medicines as potentially alcohol interactive (Table 2) [14, 20, 21, 25, 26, 28, 30–32, 34]. The potential for misclassification bias was considered high in the remaining studies as they relied on self-report for medication exposure [18, 22, 23, 27, 29, 33, 35, 36] and/or did not provide references supporting the inclusion of medicines as potentially alcohol interactive [19, 23, 24, 29, 33, 35].

While all studies relied on self-reported alcohol consumption, thus introducing potential biases in recall and reporting, 11 studies were considered to have a lower risk of bias as they reported on both quantity and frequency of alcohol consumption within a specified recall period, ranging from 1 week to 12 months (Table 2) [14, 18–23, 25, 26, 31, 34]. The risk of misclassification bias, specifically underestimating exposure to alcohol, was considered to be higher in the remaining studies as they used quantity or frequency measures alone [24, 32, 35], did not specify the recall period [24, 27, 30, 35], restricted measurements to wine consumption [29, 36] and used a very narrow recall period “last working day or last weekend” [28]. Ascertainment of exposure to alcohol was unclear in one study [33].

In relation to outcome assessment, no study directly measured the concurrent use of alcohol and AI medications, rather all studies inferred concurrent use. Although possible for all studies, the risk of misclassifying the outcome of concurrent use was considered lowest in those studies identified as having a low risk of misclassifying exposure, who used the same recall period for both exposures or inferred concurrent use based on alcohol consumption within a specific recall period, ranging between 1 week and 12 months, and current or regular medication use (Table 2) [14, 20, 21, 25, 26, 31, 34].

### Summary of findings

As noted in Table 1, all estimates relate to studies from North America, Europe and Australia.

### Alcohol consumption

The prevalence of alcohol consumption ranged between 57 and 63% in studies reporting on nationally representative samples of community dwelling older adults (Table 3) [21, 24, 27]. Prevalence estimates of alcohol consumption were generally lower in studies sampling restricted age-groups, for example estimates ranged between 33.7 and 44% for studies restricted to older adults aged >70 years [26] and >75 years [19]. Similarly, prevalence estimates of alcohol consumption were generally lower in those studies considered to have a high risk of selection bias [18, 29, 30, 32, 35]. For example, 20% of older adults registered on the Pennsylvania pharmaceutical assistance contract for the elderly reported alcohol consumption [30]. All studies reporting on gender differences identified a higher prevalence of alcohol consumption in men [19–21, 25, 29, 31, 36].

**Table 3:** Summary of results; prevalence of alcohol consumption, alcohol interactive medication use and concurrent use among older adults

Study	Prevalence of alcohol consumption in older adults (gender); Heavy/Problem drinking (gender)	Prevalence of alcohol interactive medication use:	Concurrent use reported among: Total sample of older adults	Concurrent use reported among: AI medication users	Concurrent use reported among: Current drinkers
Studies reporting on a wide range of alcohol interactive (AI) medicines (*n* = 13 studies)					
Adams 1995 [18]	47% drank alcohol in previous 6 months Heavy (>7 drinks/week): 8%	80% used one or more of the following in the last 6 months: NSAIDS, aspirin, sedatives, narcotics, antidepressants, anti-hypertensives, antacids, H_2_ blockers, warfarin & meds for congestive heart failure, gout or diabetes	38% reported concurrent use of alcohol and AI medications, 6% reported concurrent heavy alcohol consumption and AI medications		Overall drinkers: 80% used an AI medication (50% used anti-hypertensives, 27% used aspirin, 20% used NSAIDs, 18% used chronic heart failure drugs, 11% used sedatives; 5% used narcotics, 5% used warfarin, 4% used diabetic drugs, 3% used antidepressants, 3% used drugs for gout) Heavy drinkers: 80% used an AI medication (48% used anti-hypertensives, 28% used aspirin, 16% used NSAIDs, 16% used chronic heart failure drugs, 4% used sedatives, 4% used narcotics, 4% used warfarin, 8% used diabetic drugs, 12% used antidepressants, 4% used drugs for gout)
Aira 2005 [19]	44% drank alcohol in previous 12 months (66% of men & 37% of women) Heavy (>7 units/week): 7% of men & 0% of women	90% used one or more of the following regularly or as needed: acetaminophen, anticonvulsants, antidepressants, antihistamines, benzodiazepines, histamine H2 receptor agonist, neuroleptics, nitrates, NSAIDs, opiates or warfarin	39% reported concurrent use of alcohol and AI medications, 1.9% reported concurrent heavy alcohol consumption and AI medications. Concurrent alcohol and specific AI medications: 7.5% acetaminophen, 0.19% anticonvulsants, 5.16% antidepressants, 0.76% antihistamines, 11% benzodiazepines, 1.15% histamine H2 receptor agonist, 2.29% neuroleptics, 23% nitrates, 29% NSAIDs, 3.8% opiates & 3.6% warfarin	Overall AI medication users: 44% drank alcohol (36% acetaminophen users, 17% anticonvulsants users, 40% antidepressants users, 21% antihistamines users, 38% benzodiazepine users, 43% histamine H2 receptor agonist users, 25% neuroleptic users, 43% nitrate users, 46% NSAID users, 38% opiate users & 40% warfarin users)	Overall drinkers: 88% used AI medications
Breslow 2015 [20]	47% drank alcohol in previous 12 months (55% of men & 39.7% of women)	78.6% used one or more of the following: cardiovascular agents, CNS agents, coagulation modifiers, GI agents, metabolic agents, psychotherapeutic agents, respiratory agents	35% reported concurrent use of alcohol and AI medications. Concurrent alcohol and specific AI medications: 28% cardiovascular agents, 10% CNS agents (1.8% anticonvulsants, 2% anxiolytic/sedative/hypnotic, 2% narcotics, 2.2% NSAIDs), 3% coagulation modifiers, 2.2% GI agents, 16.9% metabolic agents, 3.9% psychotherapeutic agents (3.8% antidepressants), 2.1% respiratory agents	Overall AI medication users: 45% drank alcohol (44% cardiovascular agents users, 40% CNS agent users (34% anticonvulsants, 40% anxiolytic/sedative/hypnotic, 43% NSAID), 44% coagulation modifier users, 43% GI agent users, 43% metabolic agent users, 41% psychotherapeutic agent users (42% antidepressants), 48% respiratory agent users)	Overall drinkers: 77.8% took AI medications (61.3% used cardiovascular agents, 22% used CNS agent (3.9% used anticonvulsants, 4.6% used anxiolytic/sedative/hypnotic, 4.8% used NSAID), 6% used coagulation modifier, 4.7% used G I agent users, 36.5% used metabolic agent, 9.6% used psychotherapeutic agent (9.2% used antidepressants), 4.6% used respiratory agent)
Cousins 2013 [21]	62.8% drank alcohol previous 6 months (72% of men & 59% of women) Heavy (>4 drinks/day or >10 drinks/week): 20% (32% of men & 11% of women) CAGE: 8% (12.2% of men & 4% of women)	72% took one or more of the following: cardiovascular agents, CNS agents, antihistamines, Blood, antidiabetic agents, anti-infectives, GI agents, immunomodulators or muscle relaxants		Overall AI medication users concurrent use of alcohol: 60% drank alcohol (60% cardiovascular agent users, 53.5% CNS agent users (59% of NSAID users, 54% hypnotic users, 44% anxiolytic users, 52.9% antidepressant users), 66.9% antihistamine users, 58.5% blood medication users, 54% antidiabetic agent users, 47% anti-infective users, 50% GI agent users, 51% immunomodulator users &80.3% muscle relaxant users) AI medication users concurrent heavy alcohol consumption: 25% antihistamine users, 20% cardiovascular agent users, 20% blood (anti-coagulant or anti-platelet) users, 20% anti-diabetic agent users, 16% CNS agent users (13% users of anti-epileptic agents; 13% antipsychotic agents; 13% hypnotic users & 18% antidepressant users)	
Del Rio 1996 [23]	Approximately 20% drank alcohol at least once per day in the past 2 weeks	75–80% took one or more medication in previous 2 weeks	18% reported concurrent use of alcohol and AI medications		
Forster 1993 [24]	57.1% reported using alcohol 16.9% admitted to drinking enough to become “lightheaded”	Not Reported	25% reported concurrent use of alcohol and AI medications. Concurrent alcohol and specific AI medications: 19% OTC analgesics, 6.9% antihypertensives, 5.4% diuretics; 4.3% OTC cold preparations, 2.1% mind altering drugs, 1.5% diabetes pills, 1.5% prescription pain medication, 1.2% sleeping pills, 0.6% prescription blood thinners, 0.6% insulin & 0.3% seizure medications		
Immonen 2013 [27]	62.6% drank alcohol At risk drinking (>7 drinks/week, or ≥5 drinks on typical drinking day, or ≥3 drinks several times/week): 7.9%	42% took one or more of the following: metronidazole, tinidazole, disulfiram, griseofulvin, prazosin, metformin & tacrolimus. CNS agents, hypoglycaemics and warfarin		Overall AI medication users: 62.2% drank alcohol	Heavy or at risk drinkers: 42.2%% took AI medications (2.2% used antipsychotics, 4.4% used anti-depressant, 6.7% used anxiolytics, 11.1% used hypnotics/sedatives, 5.6% used anti-epileptics, 3.3% used opioids, 11.1% used warfarin & 13.3% used metformin)
Onder 2002 [29]	54.2% drank ≤40 g of wine/day prior to hospital admission (68.1% of men & 42.8% of women)	27% used diuretics, 23% digoxin, 17.7% calcium channel blockers, 16% ACE inhibitors, 15% aspirin & anti-platelets, 9% oral hypoglycaemic agents, 6% NSAIDs, 6% antibiotics, 5.2% nitrates, 5% insulin, 4% steroids & 3.2% antipsychotics			Overall wine drinkers: 26% used diuretics, 3.8% oral hypoglycaemic agents, 2.6% antipsychotics & 1.8% insulin
Pringle 2005 [30]	20.3% drank alcohol Heavy (> 30 drinks/month): 1.2%	77.4% used on or more AI medication		Overall AI medication users concurrent use of alcohol: 19% drank alcohol (18.4% cardiovascular agent users, 18% CNS agent users (20% of NSAID users, 16.8% anxiolytic/hypnotic/sedative users& 16% antidepressant users), 20% antihistamine users, 14% blood medication users, 13% antidiabetic agent users, 16% anti-infective users, 14% GI agent users & 16% muscle relaxant users)	
Qato 2015 [31]	41% were regular drinkers in the past 3 months (59.3% of men & 40.7% of women) Heavy (2–3 drinks/day): 19.7%	57.7% used at least one AI medication.	21% reported concurrent use of alcohol and AI medications		Overall drinkers (Regular drinkers): 51% used AI medications (8.4% used antidiabetic agents, 6.6% used analgesics, 2.4% used narcotics, 5.3% used acetaminophen, 26.7% used aspirin, 18.9% used psychotropic medication, 8.5% used antidepressants, 6% used anxiolytic/sedative/hypnotics & 4% used warfarin)
Swift 2007 [33]	18% drank alcohol daily in past 12 months	87.3% used at least one AI medication in last 24 h	35.4% reported concurrent use of alcohol and AI medications in previous 24 h		
Wong 2016 [35]	38% consumed alcohol Heavy (> 31 drinks/month): 6%	83% reported any medication use	31% reported concurrent use of alcohol and medications& 1.4% reported concurrent heavy alcohol consumption and medication use		
Studies reporting on psychotropic medicines (*n* = 8 studies)					
Del Rio 2002 [22]	Not Reported	13.4% used benzodiazepines in previous 2 weeks		Overall benzodiazepine users concurrent use of alcohol: 23% (56–66 years), approx. 15% (66–75 years) & approx. 10% (>75 years)	
Du 2008 [14]	47.3% drank alcohol at least once in the last-week Heavy drinking (≥10 g/day for women/ ≥ 20 g/day for men): 14.8%	20% reported use of at least one psychotropic medication.	7.4% reported concurrent use of alcohol and psychotropic medications & 2.4% reported concurrent heavy alcohol consumption and psychotropic medication use	Overall psychotropic medication users concurrent use of alcohol: 37.5%	Overall drinkers: 16% used psychotropic medication
Ilomaki 2008 [25]	76.7% drank alcohol in previous year (87.5% of men & 68.9% of women) Heavy drinking (>14 units/week men & >7 units/week women): 12.6% (16.6% of men & 7.9% of women)	11.5% reported use of at least one psychotropic medication		Overall psychotropic medication users concurrent use of alcohol: 38.9% of male users & 14.7% of female users were frequent drinkers, 25.9% of male users & 8.3% of female users were heavy drinkers	
Ilomaki 2013 [26]	33.7% drank alcohol daily in the past 12 months Heavy drinking (>2 drinks/day): 19.2% Problem drinkers (CAGE): 11%	13.6% reported use of at least one psychotropic medication (8% reported antidepressant use, 5.7% sedative or anxiolytics use & 1.6% both drug classes)		Overall antidepressant users: 27.1% consumed alcohol daily & 15% heavy drinkers Sedative or hypnotic users: 42.7% consumed alcohol daily & 26% heavy drinkers	
John 2007 [28]	Prevalence not reported Risky drinking (>20 g/day women & >30 g/day men): 15.1% of men & 3.2% of women (excludes users of sedative-hypnotic-anxiolytics and opioids)	Men: 3.8% used sedative, hypnotic, anxiolytic; 1% used opioids Women: 6.8% used sedative, hypnotic, anxiolytic; 1.6% used opioids			Men: Risk drinkers (non-smoker) use of sedative, hypnotic or anxiolytic: 4.3% of 60–79 year olds Men: Risk drinkers (smokers) use of sedative, hypnotic or anxiolytic: 0% of 60–79 year olds Women: Risk drinker (non-smoker) use of sedative, hypnotic or anxiolytic: 13% of 60–79 year olds Women: Risk drinkers (smokers) use of sedative, hypnotic or anxiolytic: 0% of 60–79 year olds
Lagnaoui 2001 [36]	56.3% drank wine (77.2% of men & 41.1% of women) Heavy drinking >0.25 l wine/day: 15.3% (32.3% of men & 3.1% of women)	32% used benzodiazepines	15.7% reported concurrent use of alcohol (wine) and benzodiazepines & 2.9% reported concurrent heavy alcohol consumption (wine) and benzodiazepine use	Overall benzodiazepine users: 49% consumed wine & 9.2% heavy drinkers	Overall wine drinkers: 28.1% used benzodiazepines & heavy wine drinkers: 5.3% used benzodiazepines
Sheahan 1995 [32]	38% drank alcohol in the past 12 months	28% reported use of at least one psychotropic medication	2% reported concurrent use of alcohol and psychotropic medication		
Veldhuizen 2009 [34]	Not reported	Not reported		Overall benzodiazepine users: 33.9% consumed alcohol & 5.1% were heavy drinkers	

### Alcohol interactive (AI) medicines

Exposure to alcohol interactive medications varied across studies; nationally representative studies of community dwelling older adults using objective measures of AI exposure estimate exposure at between 72 and 79% among the total study samples (Table 3) [20, 21]. Studies with objective measures of exposure to psychotropic medications in community dwelling older adults reported prevalence estimates of between 11.5–20% among the total study samples [14, 25, 26]. In contrast to alcohol consumption, use of psychotropic medications was significantly higher in women than men [14, 22, 25, 28, 32].

### Concurrent use of alcohol and AI medications among older adults

Eleven studies reported on the concurrent use of alcohol and alcohol interactive medications among the total study samples (Table 3) [14, 18–20, 23, 24, 31–33, 35, 36]. Eight of the 11 studies reported on alcohol consumption and a wide range of medicines with potential to interact with alcohol, with the prevalence of concurrent use ranging between 18 and 39% among the total study samples [18–20, 23, 24, 31, 33, 35]. Only two of these studies were considered to have a low risk of misclassification bias for concurrent use [20, 31] . Breslow et al. estimated the prevalence of concurrent use among older adults at 35% [20]. Concurrent use was highest for cardiovascular agents (28%) followed by metabolic agents (17%), CNS agents (10%) and coagulation modifiers (10%). Approximately 7.75% of all older adults were identified as concurrent users of alcohol and psychotropic medications [20]. Qato et al. reported a prevalence estimate of 21%, with potential alcohol-medication interactions in older adults aged between 57 and 84 years being significantly more likely among men, white respondents, wealthier respondents, and those with higher education levels and greater comorbidities [31]. The prevalence of concurrent use was lower in studies which focused on psychotropic medications, ranging between 2 and 15.7% [14, 32, 36]. Only one study was identified as having a low risk of misclassifying concurrent use in older adults [14], with an estimated prevalence of 7.4% among the total study sample and 2.4% for concurrent heavy alcohol consumption and psychotropic medications. Older age (70–79 years), residing in rural or small town areas, living alone, higher social status, polypharmacy and a poor social network were independently associated with concurrent use of alcohol and psychotropic medications [14]. While a higher proportion (15.7%) of older adults in a French study [36] were identified as concurrent users of wine and benzodiazepines, the methods of ascertaining exposure to benzodiazepines was unclear, as was the assessment of concurrent use. Similarly, the lower estimate of 2% for concurrent use of alcohol and psychotropic medications in Sheahan et al. [32] is likely an artefact of their sample including patients in congregate care facilities.

Five studies assessed the prevalence of alcohol consumption among users of a broad range of alcohol interactive medications (Table 3) [19–21, 27, 30]. Breslow [20] and Cousins [21] were identified as having a low risk of misclassifying concurrent use, and reported prevalence estimates of 45 and 60% respectively. Cousins et al. [21] found that older adults using AI medications were significantly less likely to report alcohol consumption compared to those unexposed to AI medications. Younger age (60–64 years), men, urban dwelling, higher levels of education and a history of smoking were independently associated with concurrent use of alcohol and AI medications [21]. A further six studies focused on psychotropic medication users [14, 22, 25, 26, 34, 36], four of which were identified as having a low risk of misclassification bias [14, 25, 26, 34]. Du et al. estimated the prevalence of alcohol consumption among users of psychotropic medications at 37.5% [14]. Similarly, Ilomaki et al. [25] reported that 38.9% of male psychotropic medication users, and 14.7% of their female users, consumed alcohol, with 26% of male and 8.3% of female users identified as heavy drinkers. Male psychotropic medication users consumed greater quantities of alcohol, and more often, than non-users. This pattern was not observed among women [25]. Between one-third and a half of all sedative or hypnotic users (34–54%) have been shown to consume alcohol [20, 21, 26, 34] with between 5 and 13% drinking heavily [21, 34]. Consistent with these findings, Ilomaki et al. [15] found that 26% of men aged greater than 70 years who use sedatives or hypnotics drink heavily; heavy drinking and daily drinking was significantly higher among male sedative or hypnotic users compared to non-users [26]. Concurrent use of alcohol among older adults taking antidepressants ranged between 42 and 53%, [20, 21] with 27% of male users aged greater than 70 years reporting concurrent use [26].

### Adverse outcomes associated with concurrent use of alcohol and alcohol interactive medications

Only four studies reported on adverse outcomes. Three studies reported on falls, all three studies were cross-sectional [27, 32, 35]. A study by Immonen et al. [27] of 2100 older adults in Finland found that falls and injuries when a person has consumed alcohol in the past 12 months were more common among at risk drinkers (>7 drinks/week, or ≥5 drinks drinking days or ≥3 drinks several times/week) using AI medications (13.8%) compared to AI medication-users who were not considered as at risk drinkers (4.1%) (*p* < 0.001) [27]. In contrast to these findings, Sheahan et al.’s [32] study of older adults in America, which included patients in congregate care facilities, found that although the number of psychotropic drugs was associated with an increased odds of falling, the concurrent use of alcohol and psychotropic drugs was not. Similar non-significant associations were reported in Wong et al.’s [35] convenience sample of older adults in the US. One study examined the association between moderate alcohol consumption (≤ 40 g of wine per day) and adverse drug reactions among older adults at the point of admission to one of 81 acute care hospitals in Italy [29]. Among 22,778 participants, 3.9% were identified as having one or more adverse drug reactions. Moderate alcohol consumption was associated with a 24% increase in the odds of having an adverse drug reaction [29].

## Discussion

Overall, the results of this review suggest that between one-in-five and one-in-three older adults are potentially susceptible to alcohol-medication interactions, with more than half of AI medication users reporting alcohol consumption [20, 21, 31]. However, these estimates need to be interpreted with caution as studies differed in their classification of AI medications. In the absence of an explicit list of alcohol-interactive medications, multiple drug reference sources were used across studies [20, 21, 27, 30, 31]. Use of different medication compendia led to a lack of consistency in the inclusion of AI medications across studies and may have led to the over or under inclusion of medications [31, 37], resulting in an over-or-under estimate of concurrent use. There was however consensus with regards to CNS agents; all studies that reported on AI medicines classified CNS agents as alcohol interactive, specifically psychotropic medications [14, 18–22, 24–32, 34, 36]. Based on the quality assessment of studies, the most reliable estimates for the concurrent use of psychotropic medications and alcohol ranged between 7.4% [14] and 7.75% [20].

Despite the high prevalence of concurrent use among older adults, no study examined longitudinal associations with adverse outcomes. Three cross-sectional studies reported on falls with mixed findings [27, 32, 35]; with one study reporting on an association between moderate alcohol consumption and adverse drug reactions among older adults at the point of admission to hospital [29]. An evidence based list of medications which have a significant risk of harm to older patients when combined with alcohol would be useful in a clinical setting, allowing for the identification of older adults whose alcohol consumption places them at increased risk and who would benefit from a preventative intervention. While recent studies have shown that clinicians rarely undertake screening and brief interventions to reduce alcohol consumption [38–40], flagging patients at the point of prescribing an alcohol-interactive medication may facilitate targeted screening and interventions to reduce harm. Brief alcohol interventions in primary care are effective in significantly reducing weekly alcohol consumption [41]. Additionally, educating older adults in relation to the risks associated with concurrent use of alcohol and medications has been shown to increase older adults’ awareness of potential risks [42]. However, whether this intervention results in a behaviour change for those at risk is unclear [42].

This is the first systematic review to examine the prevalence of concurrent use of alcohol and alcohol interactive medications and associated adverse outcomes in older adults. An explicit and robust methodology was applied to identify, critically appraise and synthesise the study findings. However, the findings of the review need to be interpreted in the context of the study limitations. The risk of misclassification bias for both alcohol and AI medications was high across many studies, undermining internal validity. Furthermore, no study directly measured the concurrent use of alcohol and AI medications, rather all studies inferred concurrent use. Finally, heterogeneity across studies in relation to classifying medications as alcohol interactive and in the methods used to quantify alcohol consumption, prevent statistical pooling of data from existing studies. Variation in study setting and age restrictions, also make it difficult to compare prevalence of concurrent use across studies. Furthermore, due to the current gap in the literature, the available evidence for this review was restricted to three continents, Europe, North America and Australia.

## Conclusions

While there appears to be a high propensity for alcohol-medication interactions in community dwelling older adults, there is a lack of consensus regarding what constitutes an alcohol interactive medication. An explicit list of alcohol interactive medications needs to be derived, and validated prospectively to quantify the magnitude of risk posed by the concurrent use of alcohol and alcohol interactive medications for adverse outcomes in older adults. This will allow for risk stratification of older adults at the point of prescribing, and prioritise alcohol screening and brief alcohol interventions in high-risk groups.

## Additional files

Additional file 1: Database search: this document describes the retrieval process of studies for the systematic review. (DOCX 14 kb)

Additional file 2: Search strategy: This file describes the search strategies used in Embase, PubMed, Web of Science and Scopus in order to identify studies for this systematic review. (DOCX 15 kb)

Additional file 3: Newcastle-Ottawa Scale (NOS) adapted for cross-sectional studies. (DOC 35 kb)

Additional file 4: Table S1. Most common alcohol interactive (AI) medicines across included studies. (DOCX 281 kb)

## Abbreviations

ADRs: Adverse drug reactions
AI: Alcohol interactive
AUD: Alcohol use disorder
NSAIDs: Nonsteroidal anti-inflammatory drugs
OTC: Over the counter
