# Supplementary material for: Concurrent use of alcohol interactive medications and alcohol in older adults: a systematic review of prevalence and associated adverse outcomes
Source: BMC Geriatr. 2017 Jul 17;17:148. doi: 10.1186/s12877-017-0532-2 (PMC5512950; doi:10.1186/s12877-017-0532-2)
Supplement: Supplementary file 1 — Database search: this document describes the retrieval process of studies for the systematic review. (DOCX 14 kb) [file 12877_2017_532_MOESM1_ESM.docx]

**Additional File 1: LITERATURE SEARCH**

This document describes the retrieval process of studies for the systematic review.

**DATABASE SEARCHES:**

**Electronic Databases:**

| **Date:** | **Database:** | **# records received** |
| --- | --- | --- |
| 31^st^ May 2016 | Emtree | 62 |
| 1^st^ of June 2016 | MEDLINE PubMed | 162 |
| 1^st^ of June 2016 | Web of Science | 68 |
| 1^st^ of June 2016 | Scopus | 314 |
|  | **Total** | **606** |

|  | **# records** |
| --- | --- |
| **Handsearch of articles:** | **8** |
| **Removal of duplicates:** | **68** |
| **Records after removal of duplicates** | **546** |
