# Supplementary material for: Concurrent use of alcohol interactive medications and alcohol in older adults: a systematic review of prevalence and associated adverse outcomes
Source: BMC Geriatr. 2017 Jul 17;17:148. doi: 10.1186/s12877-017-0532-2 (PMC5512950; doi:10.1186/s12877-017-0532-2)
Supplement: Supplementary file 2 — Search strategy: This file describes the search strategies used in Embase, PubMed, Web of Science and Scopus in order to identify studies for this systematic review. (DOCX 15 kb) [file 12877_2017_532_MOESM2_ESM.docx]

**Additional Document 2:**

**Search Strategy** (**Boolean operators (e.g. AND) were used during searches of the literature)**

**EMBASE SEARCH:**

Using Embase powered by Elsevier, Emtree search terms were used. The Emtree term “drug alcohol interaction” AND “aged” were searched. A freetext search was then conducted to identify any newly published articles.

**Synonyms:** aged patient; aged people; aged person; aged subject; elderly; elderly patient; elderly people; elderly person; elderly subject; senior citizen; senium

**Aged =** Used as an age indicator in human or animal studies; in humans, greater than 64 years of age

**Drug alcohol interaction**: Synonyms alcohol drug interaction; drug ethanol interaction; interaction, drug alcohol

| **Search Date:** | **Emtree Terms Searched:** | **# records retrieved** |
| --- | --- | --- |
| 31^st^ May 2016 | 'drug alcohol interaction'/exp AND 'aged'/exp | 62 |

**PUBMED SEARCH:**

**PubMed MeSH terms** were used to search MEDLINE PubMed, accessed through RCSI databases online.

**MeSH Terms:**

**Drug Interactions:** The action of a drug that may affect the activity, metabolism, or toxicity of another drug.

**Ethanol:** A clear, colorless liquid rapidly absorbed from the gastrointestinal tract and distributed throughout the body. It has bactericidal activity and is used often as a topical disinfectant. It is widely used as a solvent and preservative in pharmaceutical preparations as well as serving as the primary ingredient in ALCOHOLIC BEVERAGES.

**Aged:** A person 65 through 79 years of age.

**AGED, 80 AND OVER:** is available. Aged, 80 and over: A person 80 years of age and older.

| **Search Date:** | **MeSH terms searched:** | **# records retrieved** |
| --- | --- | --- |
| 1^st^ of June 2016 | ((("Ethanol"[Mesh]) AND "Aged"[Mesh]) AND " AND "Drug Interactions"[Mesh]))) | 146 |
| 1^st^ of June 2016 | ("Ethanol"[Mesh] AND "Aged, 80 and over"[Mesh]) AND "Drug Interactions"[Mesh] | 16 |

**WEB OF SCIENCE SEARCH:**

| **Search Date** | **Search Terms** | **# records retrieved** |
| --- | --- | --- |
| **1^st^ of June 2016** | TOPIC: (alcohol)*AND* TOPIC: (drug) *AND* TOPIC:(interaction) *AND* TOPIC: (older adult) | 68 |

**SCOPUS SEARCH:**

| **Search Date** | **Search Terms** | **# records retrieved** |
| --- | --- | --- |
| **1^st^ of June 2016** | (alcohol  AND  drug  AND  interaction  AND  older  adult) | 314 |
