# Supplementary material for: Concurrent use of alcohol interactive medications and alcohol in older adults: a systematic review of prevalence and associated adverse outcomes
Source: BMC Geriatr. 2017 Jul 17;17:148. doi: 10.1186/s12877-017-0532-2 (PMC5512950; doi:10.1186/s12877-017-0532-2)
Supplement: Supplementary file 3 — Newcastle-Ottawa Scale (NOS) adapted for cross-sectional studies. (DOC 35 kb) [file 12877_2017_532_MOESM3_ESM.doc]

**Newcastle-Ottawa Scale adapted for cross-sectional studies (Looking at prevalence of concomitant use) (Maximum 10 stars)**

**Selection:**

1) Representativeness of the sample [COMMUNITY DWELLING OLDER ADULTS]:

a) Random sample of community dwelling older adults/ nationally representative**

b) Somewhat representative of the average in the target population (includes older adults in general sample or older adults with specific cut off points for age) * (non-random sampling)

c) Selected group of users

d) No description of the sampling strategy

2) Ascertainment of the exposure (ALCOHOL CONSUMPTION – QUANTITY X FREQUENCY OPTIMAL):

a) Quantity by frequency measures **during a specified recall period** **

b) Quantity or frequency measures **during a specified recall period** *

c) No description of the measurement tool

3) Ascertainment of the exposure (AI MEDICATIONS – specifically how were medications classified as having potential to interact with alcohol):

a) Psychotropics-well known drug class to interact with alcohol and reference to the literature**

b) Pharmacy references sources or databases which flag medications having potential to interact with alcohol (any drug classes) **

c) Provide reference sources from literature to justify inclusion of medication as AI medication (any drug classes)*

d) No description of how medications were considered as having potential to interact with alcohol

4) Ascertainment of the exposure (AI MEDICATIONS – specifically how were AI medications measured SELF-REPORT WEAKEST):

a) Prescription claims data, containers, labels or prescriptions used **

b) Self-report

**Comparability –as not applicable as exposure and outcome are the same**

**Outcome:**

1) Assessment of the outcome concurrent use of AI medications with alcohol:

a) Same recall period or likely overlap (alcohol if specified specific recall and current/regular meds) and low risk of misclassification bias for either exposures**

b) Unclear whether use is concurrent

***Notes: This scale has been adapted from the Newcastle-Ottawa Quality Assessment Scale for cohort studies to perform a quality assessment of cross-sectional studies for this systematic review. For this study removed comparability assessment as not applicable to prevalence exposure and outcomes. Also- statistical test not appropriate as these studies are looking at prevalence of concomitant use of alcohol combined with medications.***
