# Supplementary material for: Concurrent use of alcohol interactive medications and alcohol in older adults: a systematic review of prevalence and associated adverse outcomes
Source: BMC Geriatr. 2017 Jul 17;17:148. doi: 10.1186/s12877-017-0532-2 (PMC5512950; doi:10.1186/s12877-017-0532-2)
Supplement: Supplementary file 4 — Most common alcohol interactive (AI) medicines across included studies. (DOCX 281 kb) [file 12877_2017_532_MOESM4_ESM.docx]

**Additional file 4: Table S1. Most common alcohol interactive (AI) medicines studied across included studies**

| **Drug class:** | **Drugs (if listed):** |
| --- | --- |
| **Central Nervous System Agents**  [[1-17](#_ENREF_1)] | Sedatives/hypnotics [[1-12](#_ENREF_1), [14-17](#_ENREF_14)]  Antidepressants [[2-4](#_ENREF_2), [8-11](#_ENREF_8), [14-17](#_ENREF_14)],  Opioids/narcotics[[2](#_ENREF_2), [5](#_ENREF_5), [8-11](#_ENREF_8), [14-17](#_ENREF_14)]  Anticonvulsants[[2](#_ENREF_2), [5](#_ENREF_5), [8](#_ENREF_8), [10-12](#_ENREF_10), [14-16](#_ENREF_14)]  Anti-psychotics[[2](#_ENREF_2), [3](#_ENREF_3), [8](#_ENREF_8), [10](#_ENREF_10), [11](#_ENREF_11), [13](#_ENREF_13), [14](#_ENREF_14), [16](#_ENREF_16)]  Anti-Migraine preparations [[2](#_ENREF_2)]  Anti-Parkinson’s medicines [[2](#_ENREF_2)]  Psychostimulants [[2](#_ENREF_2)] |
| **Cardiovascular Drugs** [[8-16](#_ENREF_8)] | Antihypertensives [[9-16](#_ENREF_9)]  [Including: ACE inhibitors [[10](#_ENREF_10), [11](#_ENREF_11), [13](#_ENREF_13), [14](#_ENREF_14)], Angiotensin II inhibitor [[10](#_ENREF_10), [11](#_ENREF_11)], Anti-adrenergics [[10](#_ENREF_10)], Beta blockers [[10](#_ENREF_10), [11](#_ENREF_11), [14](#_ENREF_14), [15](#_ENREF_15)], Calcium channel blockers [[10](#_ENREF_10), [11](#_ENREF_11), [13](#_ENREF_13), [14](#_ENREF_14)], Diuretics [[10-13](#_ENREF_10)], Alpha blockers[[11](#_ENREF_11), [16](#_ENREF_16)] and vasodilator antihypertensives[[11](#_ENREF_11), [14](#_ENREF_14)]]  Aspirin/anti-platelets [[9](#_ENREF_9), [11](#_ENREF_11), [13](#_ENREF_13), [15](#_ENREF_15)]  Congestive Heart Failure (CHF) medicines [[9](#_ENREF_9)]  Nitrates/Chest pain medications [[8](#_ENREF_8), [11-13](#_ENREF_11), [15](#_ENREF_15)]  Anti-arrhythmic/Digoxin [[10](#_ENREF_10), [13](#_ENREF_13)]  Cholesterol lowering agents/antihyperlipidemic [[10](#_ENREF_10), [11](#_ENREF_11)]  Other [[14](#_ENREF_14)] |
| **Anticoagulants [**[**8-12**](#_ENREF_8)**,** [**14-16**](#_ENREF_14)**]** | Warfarin [[8](#_ENREF_8), [9](#_ENREF_9), [15](#_ENREF_15), [16](#_ENREF_16)] |
| **Antidiabetic agents [**[**9-16**](#_ENREF_9)**]** | Insulin [[12](#_ENREF_12), [13](#_ENREF_13), [15](#_ENREF_15), [16](#_ENREF_16)]  Sulfonylureas[[15](#_ENREF_15), [16](#_ENREF_16)]  Metformin [[15](#_ENREF_15), [16](#_ENREF_16)]  Oral hypoglycaemic agents [[13](#_ENREF_13)] |
| **Gastrointestinal agents [**[**8-12**](#_ENREF_8)**,** [**14**](#_ENREF_14)**,** [**15**](#_ENREF_15)**]** | H2 antagonists [[8-10](#_ENREF_8)]  Metoclopramide [[15](#_ENREF_15)]  Prochlorperazine [[15](#_ENREF_15)]  Ulcer medication [[12](#_ENREF_12)]  Antacids [[9](#_ENREF_9)]  Cimetidine or Ranitidine[[9](#_ENREF_9)] |
| **Non-Steroidal Anti-Inflammatory Drugs (NSAIDs) [**[**8-11**](#_ENREF_8)**,** [**13-15**](#_ENREF_13)**]** | Aspirin [[15](#_ENREF_15)] |
| **Antibiotics/anti-infectives [**[**11-16**](#_ENREF_11)**]** | Metronidazole[[15](#_ENREF_15), [16](#_ENREF_16)]  Tinidazole [[16](#_ENREF_16)] |
| **Anti-histamines [**[**8**](#_ENREF_8)**,** [**10**](#_ENREF_10)**,** [**11**](#_ENREF_11)**,** [**14**](#_ENREF_14)**]** | Sedating [[11](#_ENREF_11)]  Non-sedating [[11](#_ENREF_11)] |

**References:**

1. Del Rio, M.C., C. Prada, and F.J. Alvarez, *Do Spanish patients drink alcohol while undergoing treatment with benzodiazepines?* Alcohol, 2002. **26**(1): p. 31-4.

2. Du, Y., C. Scheidt-Nave, and H. Knopf, *Use of psychotropic drugs and alcohol among non-institutionalised elderly adults in Germany.* Pharmacopsychiatry, 2008. **41**(6): p. 242-251.

3. Ilomaki, J., et al., *Risk drinking behavior among psychotropic drug users in an aging Finnish population: the FinDrink study.* Alcohol, 2008. **42**(4): p. 261-7.

4. Ilomaeki, J., et al., *Psychotropic drug use and alcohol drinking in community-dwelling older Australian men: the CHAMP study.* Drug and Alcohol Review, 2013. **32**(2): p. 218-222.

5. John, U., et al., *Sedative, hypnotic, anxiolytic and opioid medicament use and its co-occurrence with tobacco smoking and alcohol risk drinking in a community sample.* BMC Public Health, 2007. **7**: p. 337.

6. Lagnaoui, R., et al., *Benzodiazepine use and wine consumption in the French elderly.* Br J Clin Pharmacol, 2001. **52**(4): p. 455-6.

7. Veldhuizen, S., T.J. Wade, and J. Cairney, *Alcohol consumption among Canadians taking benzodiazepines and related drugs.* Pharmacoepidemiol Drug Saf, 2009. **18**(3): p. 203-10.

8. Aira, M., S. Hartikainen, and R. Sulkava, *Community prevalence of alcohol use and concomitant use of medication - A source of possible risk in the elderly aged 75 and older?* International Journal of Geriatric Psychiatry, 2005. **20**(7): p. 680-685.

9. Adams, W.L., *Potential for adverse drug-alcohol interactions among retirement community residents.* Journal of the American Geriatrics Society, 1995. **43**(9): p. 1021-1025.

10. Breslow, R.A., C. Dong, and A. White, *Prevalence of alcohol-interactive prescription medication use among current drinkers: United States, 1999 to 2010.* Alcohol Clin Exp Res, 2015. **39**(2): p. 371-9.

11. Cousins, G., et al., *Potential for alcohol and drug interactions in older adults: evidence from the Irish longitudinal study on ageing.* Bmc Geriatrics, 2014. **14**.

12. Forster, L.E., R. Pollow, and E.P. Stoller, *Alcohol use and potential risk for alcohol-related adverse drug reactions among community-based elderly.* Journal of Community Health, 1993. **18**(4): p. 225-239.

13. Onder, G., et al., *Moderate alcohol consumption and adverse drug reactions among older adults.* Pharmacoepidemiol Drug Saf, 2002. **11**(5): p. 385-92.

14. Pringle, K.E., et al., *Potential for alcohol and prescription drug interactions in older people.* J Am Geriatr Soc, 2005. **53**(11): p. 1930-6.

15. Qato, D.M., B.S. Manzoor, and T.A. Lee, *Drug-Alcohol Interactions in Older US Adults.* Journal of the American Geriatrics Society, 2015. **63**(11): p. 2324-2331.

16. Immonen, S. and K. Pitkälä, *The prevalence of potential alcohol-drug interactions in older adults.* European Geriatric Medicine, 2012. **3**: p. S134.

17. Sheahan, S.L., et al., *Psychoactive medication, alcohol use, and falls among older adults.* J Behav Med, 1995. **18**(2): p. 127-40.
